# A New Rat Study Suggests There May Be a Biologic Explanation for Higher Fentanyl Mortality in Men Than in Women

**DOI:** 10.7759/cureus.54354

**Published:** 2024-02-17

**Authors:** Robert B Raffa, Joseph V Pergolizzi, Jeanette Mathews, Michael E Schatman

**Affiliations:** 1 Pharmacology, Temple University, Philadelphia, USA; 2 Pain Management, NEMA Research, Naples, USA; 3 R&D Operations, Enalare Therapeutics, Inc., Princeton, USA; 4 Pain Medicine, New York University (NYU) Lagone Health, New York, USA

**Keywords:** rats, female, male, overdose, fentanyl

## Abstract

Could it be possible that we should give some weight to the contribution of biological differences as contributors to the greater fentanyl mortality in males than in females? Most current explanations for a sex difference are based largely on psychosocial and other non-physiologic contributions. Our recent findings suggest a biological contribution. This could have broad implications for the interpretation and prevention of fentanyl overdose deaths.

## Editorial

Opioid-induced overdose deaths, the majority involving fentanyl, were recently documented to be two- to three-fold higher in men than in women in a recent comprehensive analysis by Butelman et al. [1] (the datasets do not include information regarding transgender or non-binary persons). The findings by Butelman et al. [1] are particularly convincing because the results are consistent across states within the United States, despite differences in demographic, economic, and psychosocial variables and levels of drug misuse and drug laws. The data are supportive of prior suggestive epidemiological studies as well as observations across other countries [2-4].

To date, the popular explanations for any sex difference in human fentanyl-induced overdose mortality have been based largely on psychosocial and other non-physiologic contributions, such as men’s greater propensity for risky behaviors, post-traumatic stress disorder (PTSD), and a variety of socioeconomic and other factors [5]. Surprisingly, there is almost no literature on the possible inherent greater physiological sensitivity of males compared to females regarding fentanyl-induced mortality. Marchette et al. [6] recently reported in a comprehensive study that fentanyl produced similar changes in respiratory depression in female and male Long Evans (LE) rats, but they stopped short of testing lethal doses. We report for what we believe is the first time that the fentanyl mortality potency is greater in healthy adult male rats (eight to nine weeks of age) of two different strains, Sprague Dawley (SD) and LE, than in healthy adult female rats of the same strain and age, and that the ratio of the difference is in very close agreement with the human ratio.

We employed an up-and-down technique in order to use the smallest number of rats, which allows the determination of a 50% lethal dose (LD50) value with as few as 6 animals [7]. The number of animals, study design, and procedures were approved by the site animal care and use committee (AN-2023-S063 and AN-2023-S064). The LD50 values (mg/kg i.v.) for SD rats were 1.75 and 5.5 for males and females, respectively, and for LE rats were 1.75 and 3.3 for males and females, respectively. Thus, for both strains of rats, fentanyl-induced mortality was greater (approximately 2- to 3-fold) in male rats than in female rats. The mortality ratio in rats compares closely to that reported by Butelman et al. [1] for humans. Although an interesting finding, the interpretation of a very small rat study correlating directly to humans must be tempered accordingly. Regarding potential biological explanations, at the time of writing, there was no potential biologic explanation. But Wasilczuk et al. [8] recently reported that male mice are more susceptible to anesthetics than female mice and that the sensitivity is modulated by testosterone. The authors hypothesize that this might be related to the greater number of active neurons in the ventral hypothalamic sleep-promoting regions in male mice compared to female mice. Whether this has relevance to the difference in fentanyl sensitivity is unknown.

Our results suggest that there might be a biological contribution to the explanation(s) for greater fentanyl-induced mortality rates in men than in women. As such, they could have broad implications for the interpretation of fentanyl overdose deaths (e.g., accidental vs. intentional overdose), the identification of predisposition and sensitivity to overdose deaths, and harm-reduction strategies. We hope that these preliminary findings will prompt research on what appears to be a potentially relevant and important phenomenon.
